# Deep Learning in Plant Phenological Research: A Systematic Literature Review

**DOI:** 10.3389/fpls.2022.805738

**Published:** 2022-03-17

**Authors:** Negin Katal, Michael Rzanny, Patrick Mäder, Jana Wäldchen

**Affiliations:** ^1^Max Planck Institute for Biogeochemistry, Jena, Germany; ^2^Data-Intensive Systems and Visualisation, Technische Universität Ilmenau, Ilmenau, Germany; ^3^Faculty of Biological Sciences, Friedrich Schiller University, Jena, Germany

**Keywords:** phenology, phenology monitoring, drones, remote sensing, deep learning, machine learning, PhenoCams, herbarium specimen

## Abstract

Climate change represents one of the most critical threats to biodiversity with far-reaching consequences for species interactions, the functioning of ecosystems, or the assembly of biotic communities. Plant phenology research has gained increasing attention as the timing of periodic events in plants is strongly affected by seasonal and interannual climate variation. Recent technological development allowed us to gather invaluable data at a variety of spatial and ecological scales. The feasibility of phenological monitoring today and in the future depends heavily on developing tools capable of efficiently analyzing these enormous amounts of data. Deep Neural Networks learn representations from data with impressive accuracy and lead to significant breakthroughs in, e.g., image processing. This article is the first systematic literature review aiming to thoroughly analyze all primary studies on deep learning approaches in plant phenology research. In a multi-stage process, we selected 24 peer-reviewed studies published in the last five years (2016–2021). After carefully analyzing these studies, we describe the applied methods categorized according to the studied phenological stages, vegetation type, spatial scale, data acquisition- and deep learning methods. Furthermore, we identify and discuss research trends and highlight promising future directions. We present a systematic overview of previously applied methods on different tasks that can guide this emerging complex research field.

## 1. Introduction

Phenology is the study of changes in the timing of seasonal events, such as budburst, flowering, fructification, and senescence (Lieth, 2013). Plant phenology has received increasing public and scientific attention due to the growing evidence that the timing of developmental stages is largely dependent on environmental conditions. Temperate vegetation, in particular, shows extreme sensitivity to climate variability (Menzel et al., 2006; Schwartz et al., 2006). Phenology is directly related to climatic conditions and plays an essential role in ecosystem processes, such as carbon- and nutrient cycling. At the individual plant level, phenology has been shown to influence fitness and reproductive success (Ehrlén and Münzbergová, 2009) and thus plays a vital role in species dispersal (Chuine, 2010). Ultimately, changes in phenology can have far-reaching consequences, from affecting species dispersal and disrupting species interactions to altering the carbon cycle and in turn influencing global climate itself (Visser and Holleman, 2001; Peñuelas and Filella, 2009; Chuine, 2010; Rafferty and Ives, 2011; Piao et al., 2019). Therefore, modeling, assessing, and monitoring phenological dynamics are vital requirements to understand how plants respond to a changing world and how this influences vegetated ecosystems. While vegetation undeniably responds to environmental variability, our current understanding of phenology is limited by the extreme difficulty of documenting phenological processes usually occurring on large spatial and temporal scales.

There are different methods of monitoring phenology, e.g., (1) human visual observations usually conducted on the individual scale (Lancashire et al., 1991; Meier, 1997; Koch et al., 2007; Denny et al., 2014; Nordt et al., 2021), (2) near-surface measurements, which are carried out on a regional to local scale (Richardson et al., 2009; Brown et al., 2016; Richardson, 2019), and (3) satellite remote sensing which is applied on regional to global scales (Cleland et al., 2007; White et al., 2009; Richardson et al., 2013; Zeng et al., 2020). With the help of sensors and cameras, it has become increasingly easy to collect large amounts of phenological monitoring data, both ground-based and through remote sensing technologies. The implementation of large-scale phenology observation methods leads to a crucial paradigm shift. Data availability and data generation are no longer problems. Vast amounts of data are generated in a relatively short time at a low cost. The feasibility of phenological monitoring today and in the future depends heavily on the development of tools capable of efficiently analyzing these enormous amounts of data (Correia et al., 2020). In addition, standardized methods and metrics for data acquisition and the development of plant phenology ontologies in order to provide standardized vocabulary and semantic frameworks need to be developed for large-scale integration of heterogeneous plant phenology data (Stucky et al., 2018).

By constructing computational models with multiple processing layers and allowing the models to learn representations of data from multiple levels of abstraction (LeCun et al., 2015), deep learning (DL) techniques have gained importance in many research and application domains. Deep learning methods have been widely used for image processing in, e.g., computer vision, speech recognition, and natural language processing (Deng and Yu, 2014; LeCun et al., 2015). In ecology, deep learning is used for species identification, behavioral studies, population monitoring, or ecological modeling (Wäldchen and Mäder, 2018a; Christin et al., 2019). In earth system science, deep learning finds application in pattern classification, anomaly detection, and space- or time-dependent state prediction (Reichstein et al., 2019). Compared to traditional classification methods, deep learning models often provide higher processing accuracy when large samples for model training and testing are available. This systematic literature review explicitly focuses on studies involving deep learning for phenology monitoring and presents an overview of the methods and technologies used. We discuss possible scientific and technical advances that can further boost the potential of deep learning for monitoring phenology in the future.

We organized the remaining sections of this article as follows: Section 2 gives an overview of the current methods for monitoring plant phenology. Section 3 introduces our research questions and the methodology of this systematic review. In section 4, we present and discuss findings for each research question. We discuss trends and future directions in section 5. Section 6 concludes the review.

## 2. Plant Phenology Monitoring Methods

**Figure 1:**
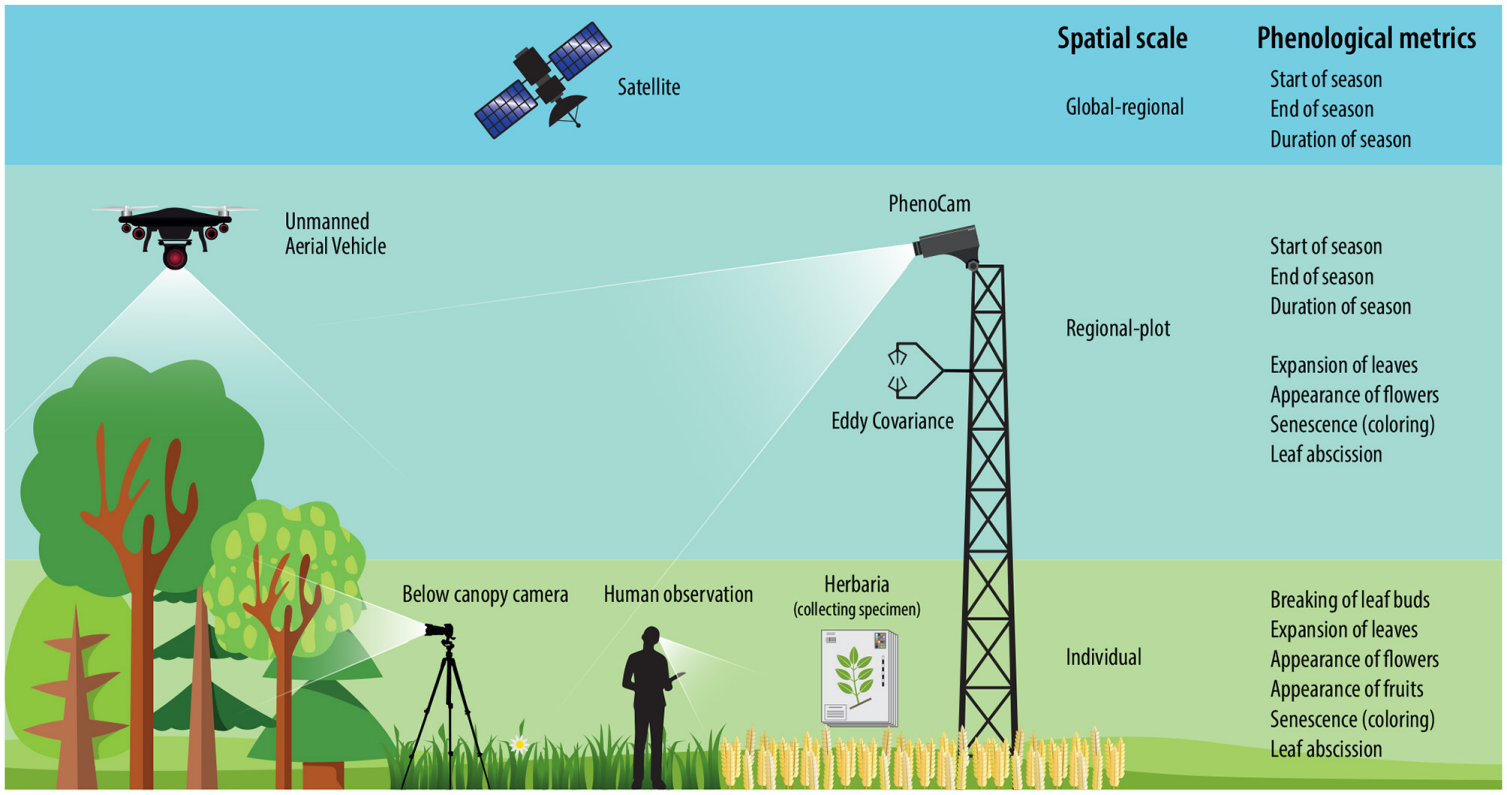
Overview of methods monitoring phenology.

Phenological stages can be detected using different methods corresponding to their spatial scale: (1) individual based observations, (2) near-surface measurements, and (3) satellite remote sensing, which are often collected across large temporal and local scales (Figure 1).

### 2.1. Individual Based Observations

Observing the date of onset and the duration of particular pheno-phases is used to quantify the phenology of individual plants, including both vegetative and reproductive events. Across different phenology monitoring projects, there is no universal definition of pheno-phases. A well-known and widely used recording scheme is the BBCH scale, which is commonly used in agricultural systems in Europe (Lancashire et al., 1991; Meier, 1997; Koch et al., 2007). Other recording schemes are the USA National Phenology Network (USA-NPN) scale (Denny et al., 2014) and the recently established scale by the PhenObs network, a phenology research initiative undertaking coordinated phenology monitoring in a network of botanical gardens distributed across many bioclimatic regions (Nordt et al., 2021). The main pheno-phases that can be found in all mentioned scales are the day-of-year (DOY) observations, e.g., breaking of leaves, breaking of buds, initial growth for annual plants, expansion of leaves, appearance of flowers, appearance of fruits, senescence (coloring), and leaf abscission (Koch et al., 2007; Morisette et al., 2009; Denny et al., 2014; Berra and Gaulton, 2021; Nordt et al., 2021).

**Human observations**. The primary method for capturing plant phenology relies on human observations of plants in phenological periods. In addition to the professional networks, such as the European Phenology Network (EPN), the USA National Phenology Network (USA-NPN) or the the PhenObs network, a number of world-wide citizen science networks strives to capture high-quality ground-based phenology observations on a voluntarily basis (Beaubien and Hamann, 2011; Taylor et al., 2019). Although these efforts are critical and acquire detailed plant phenology information at species or individual plant scale, they can typically only cover small localized areas and are limited in number.

**Below canopy cameras**. Besides cameras being installed above the canopy, capturing an entire landscape, cameras installed below the canopy are increasingly being used. These cameras can take accurate measurements on the specific individuals and record the same phenological stages as the ground-based manual recordings described above. So far, digital cameras have only been used in individual projects, and there is no larger network or association to collect an extensive image dataset of phenological stages for several species (Correia et al., 2020).

**Herbaria**. Researchers recently started to use the vast collections of plant specimens available from the world's herbaria for phenological analyses (Willis et al., 2017; Pearson et al., 2020). Pressed, preserved plant specimens collected over centuries and around the globe can serve as snapshots of plant phenology contributing to a wealth of research relating to the timing of phenological events (Jones and Daehler, 2018). Although most herbarium specimens were not collected to detect phenological stages, phenological data from specimens have proven reliable and irreplaceable for understanding plant phenology (Davis et al., 2015). The collection of phenological data from herbarium specimens is fundamentally based on the presence and absence of key reproductive or vegetative traits. Most often, the presence and occasionally the quantity of these traits are then used to score the specimens as being in a particular pheno-phase and representative of a particular phenological event (Willis et al., 2017). A comprehensive overview of how machine learning and digitized herbarium specimens can advance phenological research is given by Pearson et al. (2020).

### 2.2. Near-Surface Measurements

Phenology monitoring has been increasingly realized by remote sensing on scales comprising single research plots to regional, continental, and global scales (Cleland et al., 2007; Richardson et al., 2013), often referred to as land surface phenology (LSP). Over the last decade, ground and near-surface sensors have been increasingly used to collect data for LSP assessment purposes in place of or in complement to traditional plant phenology (Berra and Gaulton, 2021). This level of observation typically includes digital cameras, such as PhenoCams and below canopy cameras, sensing visible-light wavelengths, spectral radiometers detecting reflected radiance, continuous carbon flux measurements, and more recently cameras carried by Unmanned Aerial Vehicles (UAVs) (Berra and Gaulton, 2021). Phenology stages are typically estimated as the day of the year corresponding to the start of the season (SOS), end of the season (EOS), the peak of the season (POS) and the length of the growing season (LOS) (Yang et al., 2019, 2020; Tian et al., 2020).

**PhenoCams**. PhenoCams are near-surface digital cameras located at positions just above the canopy (Richardson et al., 2007, 2009) (Figure 1). They capture a valuable visual record of vegetation phenology across different ecosystems on landscape level, but at a spatial resolution that typically makes it impossible to discern individual plants (Reed et al., 2009). Digital cameras networks including PhenoCam, European Phenology Network (EPN), and Phenological Eyes Network (PEN) are already covering a wide range of ecosystems in the world (Richardson et al., 2013). Many of these data are now accessible online. PhenoCams detect leaf phenological events through the analysis of color changes over time. By quantifying the red, green, and blue (RGB) color channels, it is possible to estimate, for instance, leaf flushing and senescence, using the green and red channels, respectively (Keenan et al., 2014; Richardson et al., 2018).

**Unmanned aerial vehicles (UAVs)**. Beside fixed installed cameras, drones are also playing an increasing role in phenological monitoring (Candiago et al., 2015; Klosterman et al., 2018; Lee et al., 2018; Park et al., 2019; Budianti et al., 2021; Thapa et al., 2021). A drone typically carries either a standard digital camera or a multispectral camera while flying above the canopy and capturing aerial images in landscape-scale similar to satellite images but at much higher resolution. Compared to conventional aircraft, drones can be operated at a fraction of the cost, making more frequent observations feasible (Klosterman and Richardson, 2017). Additionally, using photogrammetry techniques on drone images facilitates significant advances over tower-mounted cameras. Orthomosaics or orthoimages simulate an undistorted perspective of the canopy, with a consistent spatial resolution over landscapes. Because of this feature, orthomosaics enable the identification and analysis of more significant numbers of individual organisms than is typically possible using tower-mounted camera imagery.

Overall, near-surface systems such as PhenoCams or UAVs can achieve high temporal resolution of phenological time series. They provide species-specific and/or site-level measurements and play an essential role in filling the “gap of observations” between satellite monitoring and the traditional on-the-ground phenology monitoring (Sonnentag et al., 2012; Klosterman et al., 2014).

### 2.3. Satellites Remote Sensing

Phenological timing and magnitude are frequently derived from satellite images *via* indices, such as spectral vegetation indices (VIs) (e.g., normalized difference vegetation index (NDVI) (Tucker, 1979), enhanced vegetation index (EVI) (Huete et al., 2002). Retrieved canopy variables, e.g., leaf area index (LAI) (Myneni et al., 2002), representing the seasonal dynamics of vegetation community in a pixel instead of the features of a specific plant as described above. This aggregation often disassociates the response signal of the landscape from that of the individual species, yet is essential for representing landscape-scale processes (e.g., water, energy, and carbon fluxes) in biosphere-atmosphere interaction and other models (Reed et al., 2009). Due to the requirement of repeated observations to study LSP, most satellite-based phenology studies relied on medium to coarse spatial resolution satellite sensors, such as MODIS and Landsat, or on fusing MODIS and Landsat imagery to improve the temporal and spatial resolution (Younes et al., 2021). The same phenological events assessed by PhenoCams or UAVs are typically also analyzed *via* satellite images, such as SOS, PGS, and EOS (Zeng et al., 2020). However, the large scale of these sensors limits the resolution of the data they provide, e.g., landscape heterogeneity is unresolved given the relatively coarse pixel resolutions provided by satellites sensors, potentially confounding phenological signals (Klosterman et al., 2018; Richardson et al., 2018). Further, mismatches in scale and specificity of observations between ground based and remote sensing measurements can bring difficulties in interpreting LSP metrics (Berra and Gaulton, 2021).

## 3. Methods

We performed a systematic literature review (SLR) according to Kitchenham (2004) and Pautasso (2013). We divided the whole process of the SLR into three fundamental steps: (1) defining research questions, (2) conducting a search process for relevant publications, and (3) extracting relevant data and metadata from identified publications to answer our research questions.

### 3.1. Research Questions

Our review comprises all published research in the field of deep learning methods applied to phenology stage assessment and thereby attempts to answer the following six research questions:

**RQ-1: How is the time of publication, venue, and geographical study site distributed across primary studies?**—Motivation: This question aims to retrieve a quantitative overview of the studies and the research locations.
**RQ-2: Which type of vegetation was investigated?**— Motivation: Phenology can be studied in different vegetation types, e.g., grassland, forest, shrubland, cropland. This question aims to provide an overview of the vegetation types that have been studied so far.
**RQ-3: At what spatial scale were the studies conducted?**—Motivation: Phenology can be recorded at various scales starting with direct observations on single individuals, through area-based remote sensing observations from a single research plot, to regional, continental, and global scales. This question aims to provide an overview of which spatial scales studies were carried out on.
**RQ-4: What kind of phenological expressions were studied?**—Motivation: The stages for plant phenology measurements usually include bud break, leaf expansion and maturation, flowering time, senescence (coloring), and leaf abscission for direct measurements or the start of the season and end of the season for land surface phenology measurements. The question aims to provide an overview of which phenology expressions were monitored.
**RQ-5: How were training data generated?**—Motivation: This question aims to study the utilized training data in detail, particularly the methods used to generate them.
**RQ-6: What kind of neural network architecture is used per analysis task?**—Motivation: This question aims to categorize, compare, and discuss deep learning methods applied in phenological monitoring.

### 3.2. Data Sources and Selection Strategy

In order to find relevant publications in the fields of biology, ecology and plant science, and computer science we searched the following popular databases: Web of Science, Science Direct, IEEE Explore, ACM Digital Library, and SpringerLink. We developed a two-part search string to identify relevant literature. “phenolog^*^” was used to restrict search results to phenology-specific texts, while “deep learning” was added to restrict to the specific nature of machine learning methods. We searched these terms in titles, keywords, and abstracts. We considered literature published from January 2016 until September 2021.

**Figure 2:**
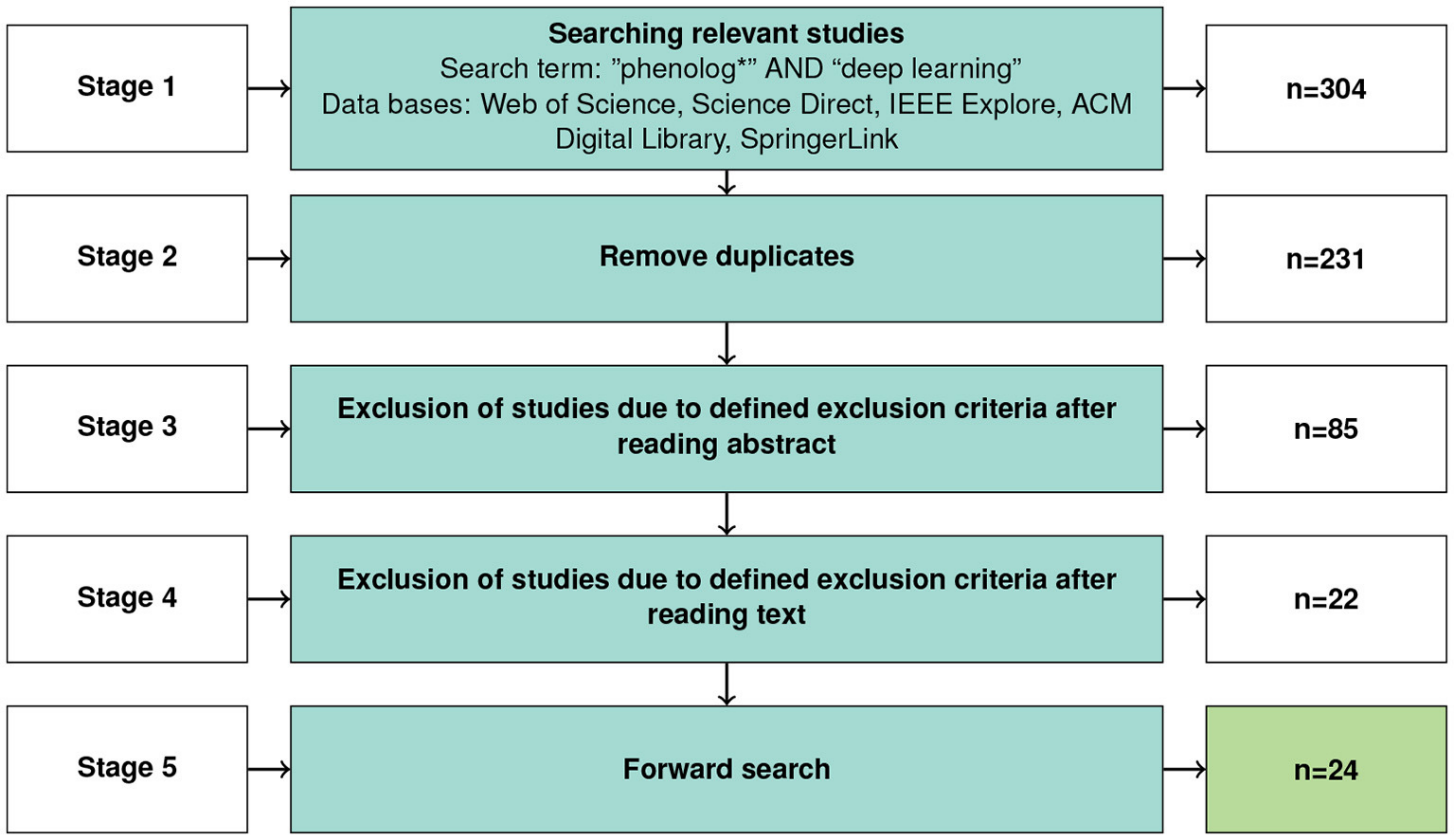
Study selection process.

An overview of the search and selection process is given in Figure 2. After an initial search a total of 304 studies were found. Due to the overlap among databases and the repeated search with similar search strings, we identified and excluded 73 duplicate matches. To further filter the relevant studies, we defined and applied the following inclusion and exclusion criteria:

- (IN): The study is published in a peer-review journal or a peer-review conference proceeding.
- (IN): The study is written in English.
- (IN): The study combined phenology research with a deep learning approach.
- (EX): The study used deep learning methods to map crop fields without using phenological features.
- (EX): The study used only shallow-learning methods, e.g., random forest or SVM for phenology research.

After a first screening of title and abstract, 146 publications were rejected, primarily due to not applying deep learning methods or not truly focussing on phenology analysis. The remaining 85 studies were analyzed in-depth, and we omitted additional 63 papers since they did not fit our IN criteria described above. In conclusion, the systematic search resulted in 22 studies. To further broaden the literature base, we added a one-step forward search starting on the studies identified by the database search based on Google Scholar citations. We checked whether the studies were listed in at least one of the five repositories in this review. Two other relevant studies were found in this search step. Eventually, the results presented in this SLR are based upon 24 primary studies complying to all defined criteria.

## 4. Results and Discussion

### 4.1. Demography and Geography of Publications (RQ-1)

**Figure 3:**
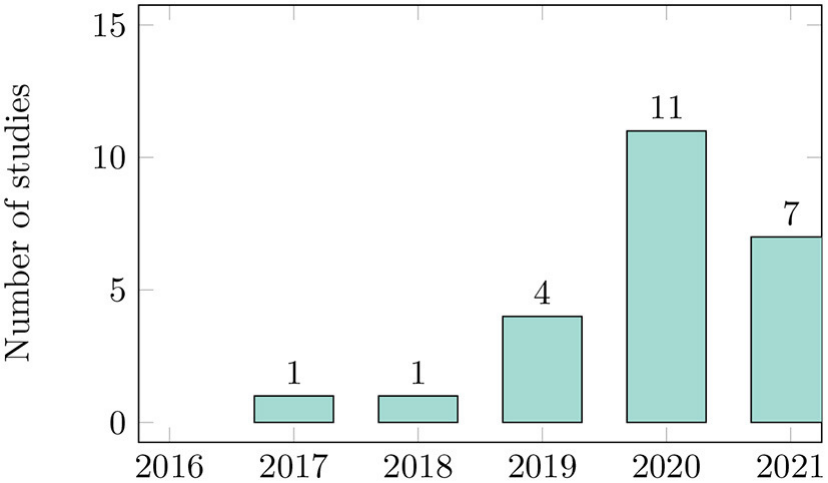
Number of studies per year of publication. In 2021, we only reviewed publications up to September 2021.

There has been a rapidly increasing interest in using deep learning for phenology research in recent years (Figure 3). The progressively rising number of published papers shows that researchers consider this research topic highly relevant. To get more insights into the geographical distribution of the study sites, we evaluated the country where each study was located. The primary studies were conducted in eleven different countries. Only the authors of one study were located in two different countries (Cao et al., 2021). Three studies have been performed in Europe; nine studies have been performed in North America, two studies in South America, six in Asian countries, and four in Australia and New Zealand. Sixteen studies were located in temperate areas, one in boreal forest, two in mediterranean areas, and two in the tropics. An overview presenting the demography and geography of the publications can be found in Supplementary Table S1. Ten of the 24 primary studies are written solely by researchers with computer science or engineering background. Ten studies were conducted in interdisciplinary groups with researchers from both fields. Four studies were written solely by ecologists. The results show that due to the steady progress in machine learning and computer vision techniques, ecologists and computer scientists are increasingly working together (Soltis et al., 2018, 2020; Wäldchen and Mäder, 2018b; Pearson et al., 2020). However, in order to apply new techniques to answer ecological questions, it is necessary to further strengthen and extend these interdisciplinary collaborations (Craven et al., 2019).

### 4.2. Vegetation Type (RQ-2)

**Figure 4:**
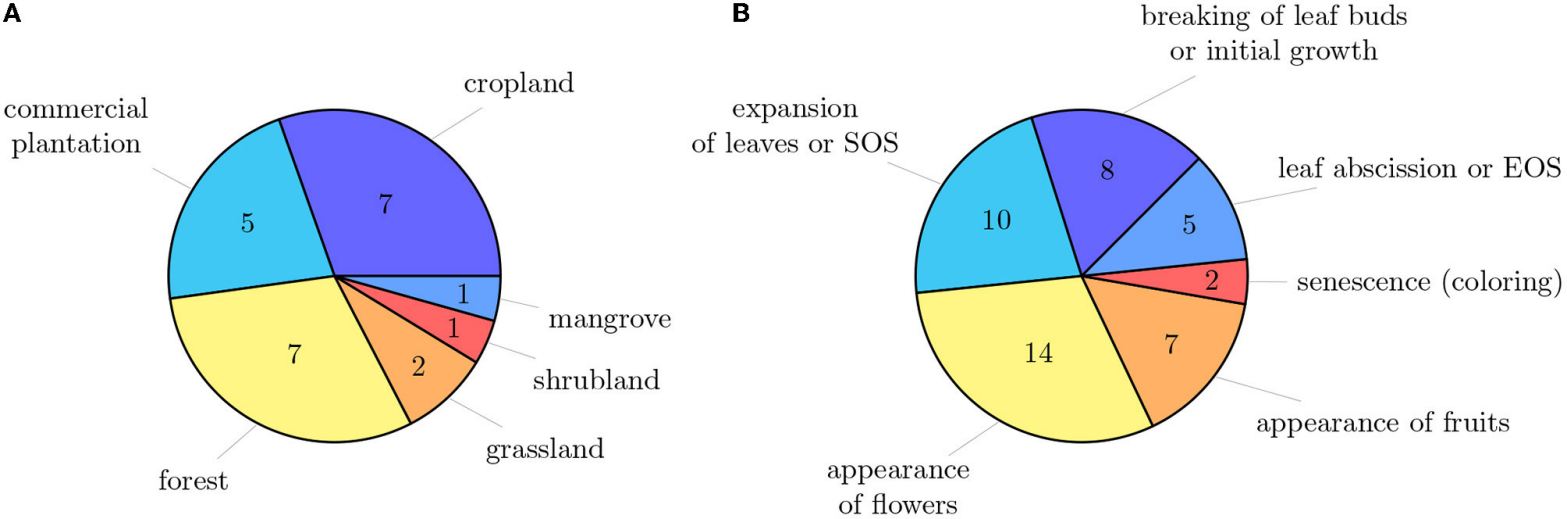
**(A)** Extent of the investigated vegetation types across primary studies. Studies that used herbarium materials are not included. **(B)** Overview of main phenological stages and the number of studies that investigated them. Some studies investigated several phenological stages.

The primary studies were conducted across different vegetation types, i.e., grassland, forest, shrubland, and agricultural land (cp. Figure 4A). Thereby, croplands (seven studies), commercial plantations (five studies), and forests (seven studies) were the most studied vegetation types. Two studies were conducted in grasslands, of which one study focused savanna-like vegetation and the other study studied savanna-like vegetation plots, as well as managed grasslands. Three studies used herbarium specimens and, therefore, adding them into the specific vegetation type category was impossible. Automated phenology stage classification is primarily being promoted in the agricultural sector. Also, it has mainly economic reasons because, e.g., the timing of weed control or harvesting depends on phenology. Additionally, crops usually grown in monocultures make croplands and commercial plantations an exciting target for studying phenology. Focusing on a specific species is more straightforward in building the training data set and evaluating the image data.

### 4.3. Observational Scale (RQ-3)

As discussed in section 2, phenological events can be recorded at different observational scales, i.e., individual scale, plot scale, regional scale, and global scale. The vast majority of the primary studies examine phenological stages on single individuals. Ten studies explored phenology on a regional level. No single study operates on a global level. Therefore, deep learning is primarily intended to simplify the very time-consuming and cost-intensive direct phenological measurements so far. In the long term, it will be possible to automatically generate more *in-situ* data over a wide geographical range and complement the manual observations by humans. In addition, it will become increasingly important to combine phenological monitoring methods at different observational scales. However, mismatches in scale and precision of observations between ground based and remote sensing measurements complicates the interpretation of phenological metrics (Berra and Gaulton, 2021). With the automation of data acquisition at different scales, it will become even more important to develop unified metrics and ontologies (Stucky et al., 2018).

### 4.4. Phenological Stages (RQ-4)

Different phenological stages were recorded for phenology studies. The main phenological stages are the breaking of leaf buds or initial growth, expansion of leaves or SOS, the appearance of flowers, appearance of fruits, senescence (coloring), leaf abscission, or EOS (cp. Figure 4B). More than half of the studies focused either on the expansion of leaves (SOS) or on the flowering time. Ten studies recorded several phenological stages during the year. However, most studies record only a single phenological stage, which leads to the fact that we hardly have intra-annual time series of the same individual, but only at a specific phenological time. Autumn pheno-phases, such as leaf coloration and leaf fall, have received considerably less attention compared to their spring counterparts, i.e., budburst and leaf unfolding, but are equally important determinants of the duration of the growing season and thus have a controlling influence on, e.g., the carbon-uptake period.

### 4.5. Training Data (RQ-5)

**Figure 5:**
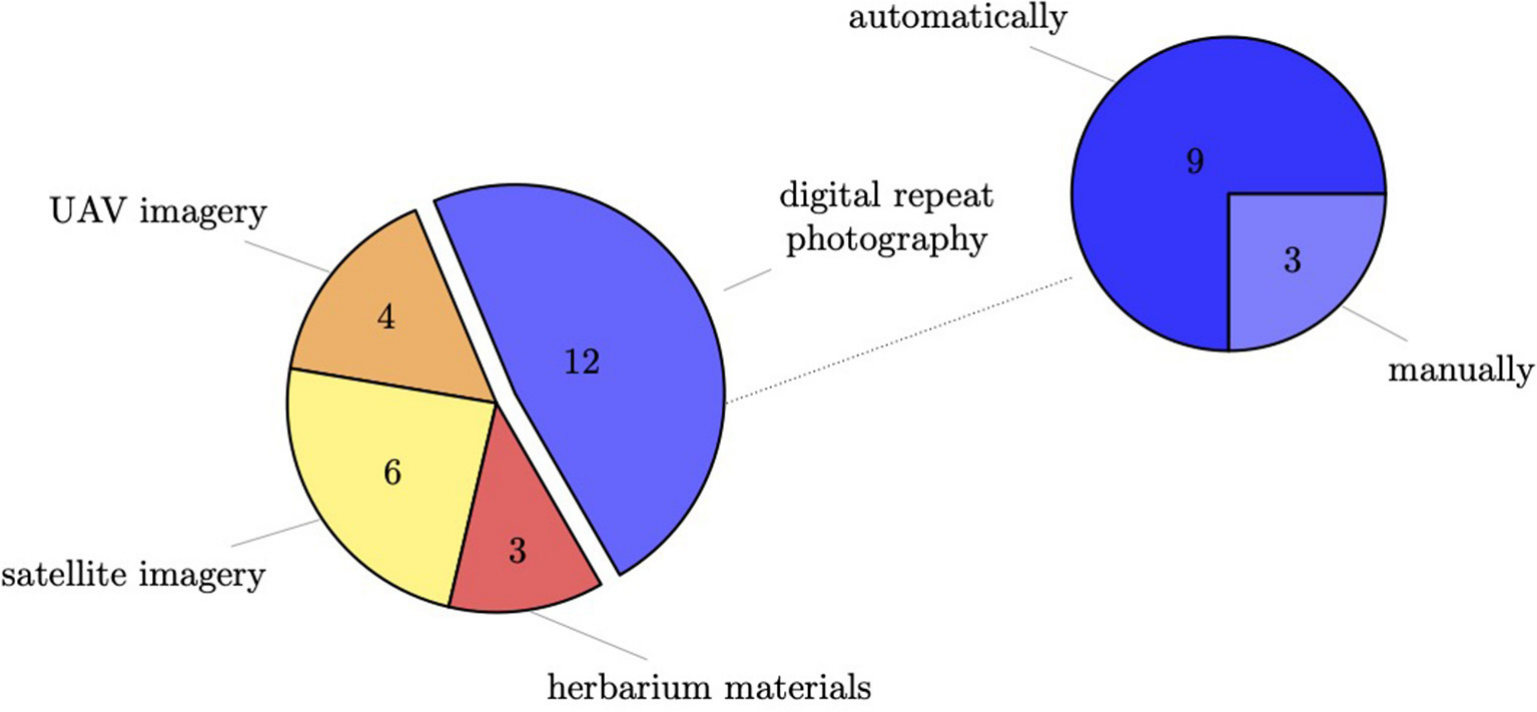
Utilization of different methods for acquiring training data across primary studies.

To classify phenological stages automatically with the deep learning approach, a large amount of training material is needed. Across the primary studies, different methods were used to acquire these training materials (cp. Figure 5). Most studies used digital repeat photography. Only one study combined different methods and used digital repeat photography and UAV images but not for the same landscape.

In total, twelve studies used images from digital repeat photography and analyzed those with deep learning methods. More specifically, three studies used handheld cameras where users manually capture images, while eight of the primary studies used automated, repeated image capturing systems. In general, studies were very diverse with respect to the way of installation and the chosen camera model. Table 1 provides a comparative overview of all the studies in terms of training data.

**Table 1:** Overview of studies used digital repeat photography for phenological study with DL methodology.

	**Vegetation** **type**	**Phenology expression**	**Image acquisition**			
**Primary study**			**Perspective**	**Scale**	**Trigger**	**# Images**
Correia et al. (2020)	Forest	Bud	Under canopy	Individual	Automated	47,607
Kim et al. (2021b)	Forest	Flower	Under canopy	Individual	Automated	20,000
Cao et al. (2021)	Forest	Leaf	Above canopy	Regional	Automated	14,453
Milicevic et al. (2020)	Plantation	Flower	Under canopy	Individual	Automated	7,000
Ganesh et al. (2019)	Plantation	Fruit	Under canopy	Individual	Manual	–
Wang et al. (2020)	Plantation	Flower	Under canopy	Individual	Automated	–
Wang et al. (2021)	Plantation	Flower	Under canopy	Individual	Automated	1,126
Pahalawatta et al. (2020)	Plantation	Flower	Off-site	Individual	Manual	245
Velumani et al. (2020)	Cropland	Wheat spike	Above crop	Individual	Automated	40,500
Yalcin (2017)	Cropland	9 stages	Above crop	Individual	Automated	2400
Han et al. (2021)	Cropland	10 stages	Above crop	Individual	Manual	610
Nogueira et al. (2019)	Grass-/shrubland	SOS	Above canopy	Regional	Automated	432

**Figure 6:**
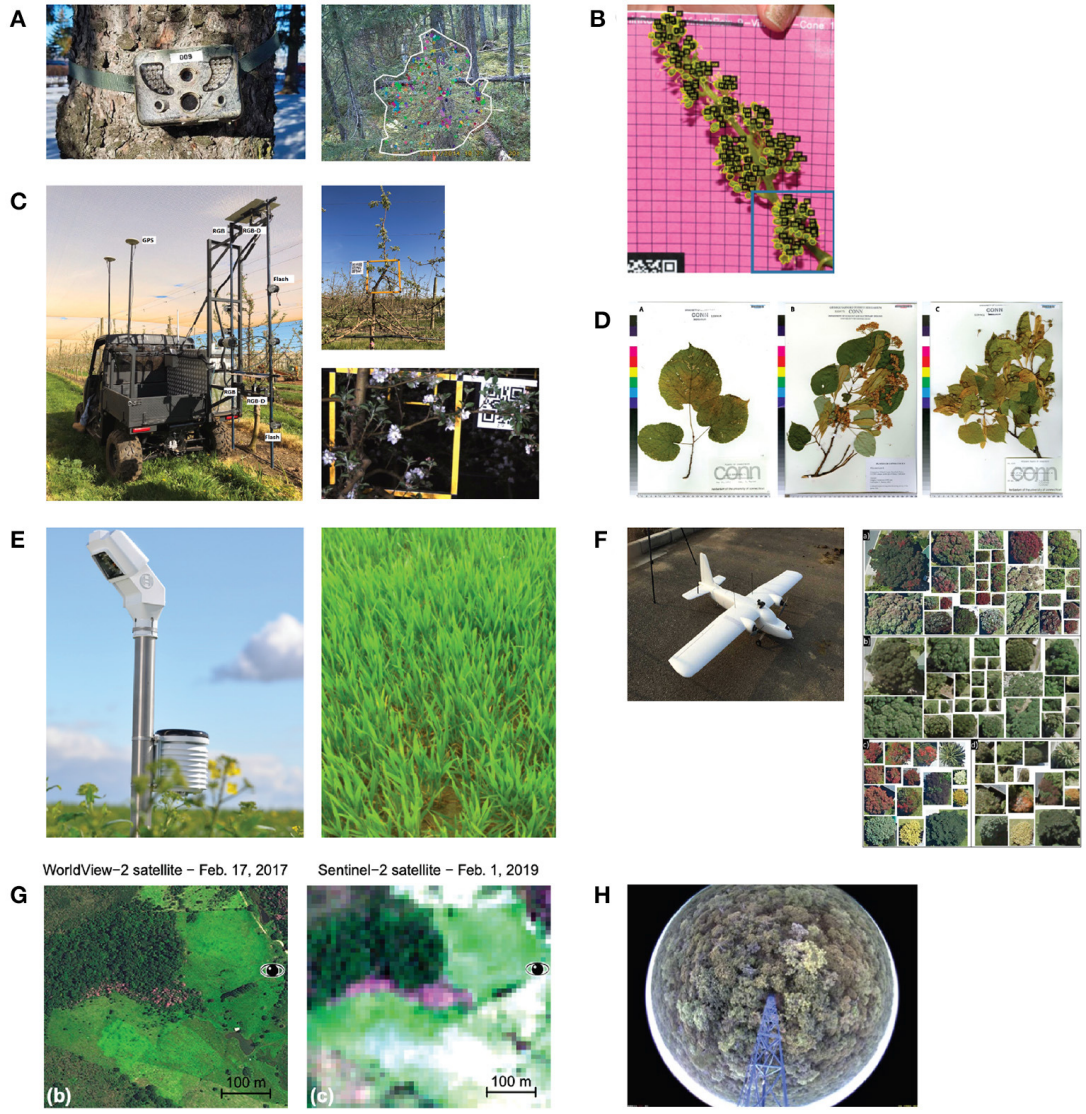
Examples of image capturing methods. **(A)** A wildlife camera was used to capture images for budburst classification in coniferous forests (Correia et al., 2020). **(B)** Images were taken manually on a plain background (Pahalawatta et al., 2020). **(C)** Images of apple flowers were collected by a mobile platform (Wang et al., 2020). **(D)** Digitalized herbarium specimens (Lorieul et al., 2019). **(E)** Close-up shots of certain areas of agriculture were taken automatically (Yalcin, 2017). **(F)** Aerial images were taken by high resolution UAVs imagery (Pearse et al., 2021). **(G)** Sentinel-2 and Worldview-2 satellite images (Wagner, 2021). **(H)** Camera installed on an 18 m tower using digital timestamps (Nogueira et al., 2019).

**Digital photography under canopy**. Correia et al. (2020) used a wildlife camera on three sites in Canada to capture budburst time in black spruce (*Picea mariana*) and balsam fir (*Abies balsamifera*) forest stand. They installed the cameras before the growing season, when budburst usually occurs, horizontally under the tree canopy onto nearby trees with approximately 5 m distance from each other. The cameras took RGB images every 30 min during daytime (cp. Figure 6A). Milicevic et al. (2020) had a similar setting for classifying the stage of flower development in an olive orchard in southern Croatia. They also acquired images in an automated manner during springtime at a distance of 40–50 cm from the tree canopy. Kim et al. (2021b) used fixed cameras installed under the canopy to identify the presence of flowers on different forest sites in Seoul's national university forests in South Korea. The investigated forest stands were dominated by broadleaved trees. They did not mention precisely how they installed the cameras, but their images show trees horizontally captured under the canopy. In contrast to the previously mentioned studies, they used the images only as validation and test dataset. The training dataset was acquired from web sources, and binary labels had been assigned indicating whether a depicted tree is in bloom or not. Wang et al. (2020) proposed an autonomous apple flower mapping system in which the image data were collected by a mobile platform automatically. Cameras were installed approximately perpendicular at 2.2 m from the ground to capture the entire canopy. The vehicle traveled forward at approximately 5 km/h with the camera facing the trees to capture images every 0.5 m (cp. Figure 6C). All data were collected within one day. In a second study, Wang et al. (2021) used the same method for data acquisition, but data were collected not only on one but throughout 26 days to classify different flower phenology stages and their distribution on the tree. The flowering stage was also investigated by Pahalawatta et al. (2020). They manually collected images during the flowering season of grapes. In contrast to all other studies, pictures were taken on monochrome background (cp. Figure 6B) and not in natural environment. Ganesh et al. (2019) tried to determine the time of fruiting automatically. They used a dataset of orange fruit images, which were taken manually with a digital camera just ahead of the commercial harvesting season. There was no further information on how the pictures were taken, but the authors report that about 60 oranges are depicted on one image.

**Digital photography above canopy**. Cao et al. (2021) was one of the pioneers in using phenocam images for leaf phenology prediction with the deep learning (DL) approach. They installed digital cameras in 56 sites in the northeastern United States and Canada in the deciduous broad-leaved forest. Each digital camera took one image every 30 min between 11:30 to 13:30. A second study by Nogueira et al. (2019) used pictures taken from a tower above the tree canopy (cp. Figure 6H). They collected a dataset with a near-remote phenological system composed of a camera set up in an 18 m tower in a savanna-like vegetation in Brazil. The camera was configured to automatically take pictures every hour over 36 days between August and October 2011. The study aimed to identify different plant species by their different greening times.

**Digital photography on croplands**. Image acquisition methods applied on cropland differ from those applied on forests or tree plantations. Yalcin (2017) collected an image dataset consisting of automatically taken close-up shots of some regions of agricultural fields every 30 min by 1,200 smart ground stations all over Turkey (cp. Figure 6E). They classified nine different phenology stages. Velumani et al. (2020) selected four different sites in the north and southeast of France to install 47 stationary sensor arrays. These sensor arrays consist of a telescopic pole installed vertically, equipped with an RGB camera 1 m above the top of the crop and additional meteorological sensors. The RGB cameras took one image per day at solar noon over three years. The goal of their study was to automatically decide whether spikes are present. Han et al. (2021) used handheld digital cameras to take images manually and classify ten different growth stages automatically. They took images during daytime from July to November 2018. All images were taken at the height of 1.5 m from four vertical and three horizontal directions.

The analyzed studies show that cameras mounted directly in the canopy can provide images that can make individual-based phenological observations in forests and plantations. So far, there is no extensive network of these installations spanning entire regions. However, we can expect such imaging schemes to increase in the future. As digital cameras become cheaper each year, and machine learning (ML) methods make it easier to analyze this amount of imagery, these types of installations will provide important phenological data alongside canopy cameras in the future, supplementing more expensive manual surveys by experts or citizens scientists. They will also become, in particular, more important in areas where it is difficult to access and regularly sample.

**Herbarium specimen**. Three of the primary studies used herbarium specimens in combination with deep learning to classify phenology stages (cp. Table 2). Lorieul et al. (2019) used four digitized specimen datasets from American herbaria (in total, 163,233 herbarium specimens belonging to 7,782 species) for automated annotation of phenology stages on herbarium specimens (cp. Figure 6D). They first tested the ability of deep learning techniques to recognize fertile material on the specimen. Second, they determine whether it is a flower or a fruit. A third experiment dealt with the automated assessment of nine different predefined phenology stages. Davis et al. (2020) used more than 3000 herbarium specimens from six common wildflower species of the eastern US to count reproductive structures such as buds, fruits, and flowers. Goëau et al. (2020) used 21 herbarium specimens of *Streptanthus tortuosus* from the Brassicaceae family to automate the detection, segmentation, and classification of four reproductive structures (flower buds, flowers, immature fruits, and mature fruits). All three studies demonstrated success in automating the collection of large amounts of phenology-relevant data from herbarium specimens with DL technologies.

**Table 2:** Overview of studies that used herbarium materials for phenological study with DL methodology.

**Study**	**Phenology expression**	**Scale**	**# Specimen**	**# Species**
Lorieul et al. (2019)	Bud, flower, fruit, sporangia, cones	Individual	163,233	7,782
Davis et al. (2020)	Bud, flower, fruit	Individual	>3,000	6
Goëau et al. (2020)	Bud, flower, fruit	Individual	21	1

**UAV imagery**. In total, four primary studies used drone images (cp. Table 3). In two studies, the aim of the investigations was to test the potential to utilize phenology to enhance species identification in RGB aerial imagery. One study estimated the rice grain yield in specific phenological stages. Only in one study specific phenological stages were detected. In Nogueira et al. (2019) images were taken between October 2015 and February 2017, one timestamp (total 15 images). These timestamps were mosaicked into a single orthoimage for spatio-temporal vegetation pixel classification. The goal of the study was to identify species by their different greening times. Pearse et al. (2021) also acquired a large orthoimage. They collected aerial imagery over Tauranga, New Zealand in summer and autumn. Here, the presence of pohutukawa (*Metrosideros excelsa*) was detected by its red flower (cp. Figure 6F). Yang et al. (2019) and Yang et al. (2020) used a fixed-wing UAV with one RGB camera and one multispectral camera to take images from different rice crop fields. In Yang et al. (2019) rice grain yield was estimated at the ripening stage using deep learning technology. In the second study Yang et al. (2020) propose an approach which identifies eight principal growth stages of rice according to the BBCH scale (Lancashire et al., 1991) and the harvest time directly from RGB images with DL. The extraction of phenological information from drone imagery using DL technology has rarely been performed. There is still a need for more research in this area, as drones have been used more and more for ecological purposes in recent years (Dalla Corte et al., 2020; Corcoran et al., 2021; Mohan et al., 2021).

**Table 3:** Overview of studies that used UAVs imagery for phenological study with DL methodology.

**Study**	**Vegetation type**	**Phenology expression**	**Scale**	**Sensor**
Pearse et al. (2021)	Forest	Flower	Individual	RGB
Nogueira et al. (2019)	Grass-/shrubland	SOS	Regional	RGB
Yang et al. (2019)	Cropland	6 growth stages	Regional	RGB, multispectral
Yang et al. (2020)	Cropland	8 growth stages + Harvest time	Regional	RGB, multispectral

**Satellite imagery**. Six primary studies used satellite imagery in combination with deep learning (CP. Table 4). Tian et al. (2020) used Landsat multispectral imagery to analyze the phenology of the invasive species *Spartina alterniflora* to predict its occurrence in the Beibu Gulf, located in the northwestern South China Sea. Here, two key phenological periods (green and senescence) of *Spartina alterniflora* were identified by analyzing the NDVI index. Extracted phenology features were then used in combination with deep learning to detect *S. alterniflora*. Cai et al. (2018) used Landsat multispectral images to calculate different phenology-related vegetation indices on croplands with the aim to identify different crop species using DL technologies. Also, Li et al. (2020) tried to classify different crop types based on different phenological developmental times using Sentinel-2 and Landsat-8 satellite in combination with different vegetation indices. Kim et al. (2021a) used satellite images from Sentinel-2 to identify deforested areas in North Korea using the NDVI index in combination with DL. Wagner (2021) used satellite imagery to predict the occurrence of in the *Pleroma spp*. in the entire Brazilian Atlantic Forest (cp. Figure 6G). Here the magenta-to-deep-purple blossoms of the trees are used for the classification tasks. Only Xin et al. (2020) used satellite data in combination with DL technologies to retrieve phenology metrics. They detected SOS and EOS of deciduous broadleaf, evergreen broadleaf, drought-deciduous broadleaf, and graminoid forests in the USA. The primary aim of most studies using satellite data was not to classify phenological stages but to classify the presence of plant species according to species-specific phenological characteristics. Only one study retrieved phenology metrics with DL. The extraction of phenological information from satellite images with DL technology is still underrepresented and shows an open research field.

**Table 4:** Overview of studies that used satellite imagery for phenological study with DL methodology.

**Study**	**Vegetation type**	**Phenology expression**	**Scale**	**Sensor**
Tian et al. (2020)	Mangrove	SOS, senescence	Regional	Landsat-5, 8
Cai et al. (2018)	Cropland	SOS	Regional	Landsat-5, 7, 8
Li et al. (2020)	Cropland	SOS, EOS	Regional	Sentinel-2, Landsat-8
Xin et al. (2020)	Forest, Grassland	SOS, EOS	Regional	MODIS
Kim et al. (2021a)	Forest	SOS	Regional	Sentinel-2
Wagner (2021)	Forest	Flower	Regional	Sentinel-2

### 4.6. Deep Learning Methodology (RQ-6)

**Figure 7:**
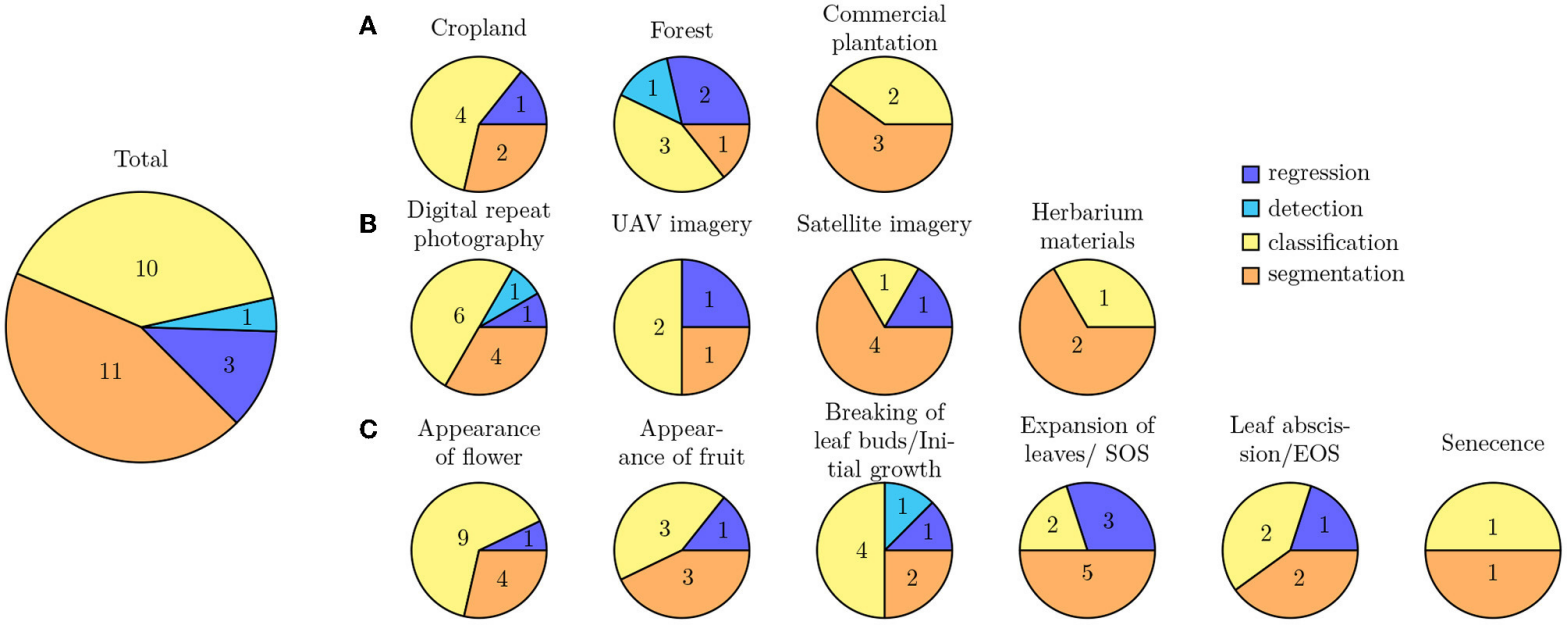
Overview of all DL methods used for different types of **(A)** landuse, **(B)** image origin, and **(C)** type of phenology expression under study.

Machine learning methods are often categorized depending on the type of task the trained model shall solve. Thereby, one categorization is based on the output a model predicts and therefore distinguishes: (1) classification tasks aiming to predict categorical class labels, e.g., the phenological stage of a plant, from (2) regression tasks aiming to predict continuous values, e.g., the amount of biomass depicted on an image (Goodfellow et al., 2016). Another categorization is based on the model's input type and the performed analysis thereof. Among the primary studies, only image data have been used as input to the trained models and the applied deep learning techniques are used to solve computer vision tasks. These techniques are categorized into: (1) image classification approaches that predict a categorical label based on the entire contents of an image, (2) object detection approaches that first predict the location of sought objects, e.g., flowers or leaves, within an image and then subsequently classify a categorical label for only the contents of this region, and (3) object segmentation approaches that predict a categorical class label for each pixel within an image, e.g., foreground and background, and thereby allow prediction of fine-grained, pixel-precise masks separating the sought objects from the background (Goodfellow et al., 2016). We observed some inconsistencies in the usage of this terminology across the primary studies and scientists with different backgrounds. For example, several studies perform a classification from a machine learning perspective but refer to it as detection. Below, we categorize all primary studies based on the machine learning task they solve into four groups: image classification, object detection, object segmentation, and regression. Figure 7 provides an overview of all DL methods used for different types of landuse, image origin, and type of phenology expression under study. Classification and segmentation methods are most frequently applied to all types of studies.

**Classification approaches**. Convolutional neural networks (CNN) are the most common approach to computer vision tasks since they allow to effectively deal with high input dimensionality of images while preserving the spatial relationship of features depicted in these images (Gu et al., 2018). A CNN is a deep neural network inspired by the organization of the natural visual cortex and designed to automatically and adaptively learn spatial hierarchies of features, i.e., a cascade of patterns where lower level features are used to compose higher level patterns. A CNN model is typically composed of three core layer types: convolution, pooling, and fully connected layers. The first two, convolution and pooling layers, perform feature extraction, and the fully connected layer typically maps the extracted features to the network's output and thereby performs the actual classification. Numerous CNN architectures for classification tasks have been proposed in the last decade, with examples of seminal architectures being: AlexNet, VGG, ResNet, Inception, and more recently EfficientNet (Raghu and Schmidt, 2020). These CNNs differ from each other in terms of their layers, their parameterization and other individual concepts, e.g., residual connections, inception blocks, and batch normalization. Ten primary studies used deep learning methods to perform classification tasks (cp. Table 5), such as the presence of certain indicator species, the presence of flowers, open vs. closed flowers, and differentiating phenological stages. Five primary studies systematically compared the performance of deep learning methods with traditional shallow learning methods or vegetation indices. All found that deep learning methods consistently outperformed traditional methods. Han et al. (2021) present the only study where a CNN was composed of four branches to process images from different perspectives separately. They concatenate the four feature vectors retrieved from the four individual perspectives and use an eventually fully connected layer to gain an overall classification score. Among other alternatives, i.e., early fusion and score level fusion, late fusion is often yielding the best performance (Rzanny et al., 2019; Seeland and Mäder, 2021). Yang et al. (2020) proposed a multi-modal analysis of images and accompanying temperature readings and fused the individual features closed to the network's output to classify phenological stages on croplands. Wagner (2021) is the only study that used satellite data for a classification task. For this investigation blossoming *Pleroma* trees were mapped using Sentinel-2. The tiles were split in images of 1.28 km length per side. Each image was classified whether it contained the blossoming *Pleroma* trees or not. The authors chose the classification method over the semantic segmentation method (more details below) for two main reasons. First, it enabled the manual production of a training sample relatively quickly. Second, binary classification is more straightforward and less computationally intensive, hence drastically reducing the time of processing and subsequent analysis (Wagner, 2021).

**Table 5:** Overview of deep learning methods in classification tasks.

	**Classification**	**Network**	**Compared**	**Performance as**
**Primary study**	**task**	**architecture**	**methods**	**accuracy**
Han et al. (2021)	10 stages	**AlexNet** (fusing 4 perspectives)	GCC, color features+SVM	**91.2**, 73.1, 81.7
Yalcin (2017)	9 stages	AlexNet	Texture feature + Naive-Bayes	**87.1**, 82.4
Yang et al. (2020)	8 stages + harvest time	**Cust. two-branch CNN processing images and temperature**	Vegetation indices, VGG16, InceptionV3, ResNet50V2, InceptionResNetV2	**83.9**, 68.8, 83.2, 81.3, 83.8, 81.3
Lorieul et al. (2019)	Fertile material	ResNet50	–	96.3
Lorieul et al. (2019)	Flower	ResNet50	–	84.3
Lorieul et al. (2019)	Fruit	ResNet50	–	80.5
Lorieul et al. (2019)	9 stages	ResNet50	–	43.4
Wang et al. (2021)	8 flower stages + distribution	**VGG16**	YOLOv5	–
Kim et al. (2021b)	Flower	**NASNet**	VGG16, ResNet50, ResNet101, MobileNet	**99.9**, 99.0, 99.2, 99.4, 98.6
Milicevic et al. (2020)	Open - closed flower buds	**Cust. CNN**	VGG19, InceptionResNetV2, Xception, ResNet50,	**97.2**, 69.5, 65.5, 67,0, 64,0
Velumani et al. (2020)	Wheat spikes	ResNet50	–	98.5
Wagner (2021)	Species presence	VGG16	–	99.6
Pearse et al. (2021)	Species presence	**ResNet50**	Texture feature+XG Boost	**97.4**, 86.7

*The bold values indicate the best performing method and their respective performance*.

**Object detection approaches**. While image classification operates on the entire image and aims to assign one or multiple labels (aka classes), object detection first aims at locating objects of interest within an image and then assigning labels to each identified object encircled by a bounding box. Object detection approaches fall into two categories: (1) two-stage approaches, such as R-CNN, and (2) one-stage approaches, such as YOLO and RetinaNet, with the former being more precise and the latter being more computationally efficient. Only a single primary study used an object detection method. Correia et al. (2020) used a RetinaNet to identify and localize multiple open buds per image from time-lapse digital photography in black spruce and balsam fir dominated forests. RetinaNet was chosen due to its outstanding detection performance with dense and small-scale objects (Lin et al., 2017), which is a good match for the small buds analyzed in this study.

**Image segmentation approaches**. Segmentation reaches the deepest possible level of detail in every single image pixel. Image segmentation is the process of classifying each pixel in the image as belonging to a specific category. This is why the output of the segmentation approach is not a set of class labels or bounding boxes, but a classification for each pixel of an image (Raghu and Schmidt, 2020). Although there are several image segmentation methods, two types of segmentation are predominant in the domain of deep learning. These are semantic segmentation and instance segmentation. Semantic segmentation performs pixel-level labeling with a set of object categories (e.g., land use categories or land cover in remote sensing data) for all image pixels (Goodfellow et al., 2016). Thus it is generally a more complex task compared to image classification, which predicts a single or multiple labels for an entire image. Instance segmentation extends the scope of segmentation further by detecting and delineating each object of interest in the image (e.g., partitioning individual flowers or fruits). Prominent semantic segmentation architectures are fully convolutional networks (FCN), and the more advanced U-Net consisting of a fully convolutional pipeline initially encoding the image *via* convolution operations before upsampling the desired mask *via* up-convolution operations. Mask R-CNN is popular deep learning instance segmentation technique that performs pixel-level segmentation on detected objects. The Mask R-CNN algorithm can accommodate multiple classes and overlapping objects. Ten primary studies focused on segmentation tasks related to phenology analysis (cp. Table 6). Semantic segmentation was mainly used for analyzing remote sensing data from satellites (Cai et al., 2018; Li et al., 2020; Tian et al., 2020; Kim et al., 2021a). The principal advantage of using CNNs in remote sensing is their accuracy, which is similar to human-level classification and detection accuracy and enables rapid application over vast areas and through time (Brodrick et al., 2019). All remote sensing studies analyzed multispectral data with different time stamps to segment species or land use categories based on species-specific phenological features. Instance segmentation, on the other hand, has been used, e.g., for the analysis of herbarium specimens (Davis et al., 2020; Goëau et al., 2020) to count phenological characteristics such as fruits or flowers. Mask-RCNN was used in all studies applying instance segmentation.

**Table 6:** Overview of deep learning methods in segmentation tasks.

**Primary study**	**Segmentation task**	**Network architecture**	**Performance**
Wang et al. (2020)	Flower segmentation	Cust. FCN	84.4 (F)
Davis et al. (2020)	Counting buds, flowers, fruits	Mask R-CNN	92.0 (A)
Goëau et al. (2020)	Counting buds, flowers, fruits	Mask R-CNN	77.9 (A)
Ganesh et al. (2019)	Fruit segmentation	Mask R-CNN	88.7 (F)
Pahalawatta et al. (2020)	Open/close flower	Mask R-CNN	84.3 (A)
Kim et al. (2021a)	Landuse	U-Net	75.0 (A)
Li et al. (2020)	Species presence	Temp. group attention netw.	99.9 (A)
Tian et al. (2020)	Species presence	SAE	96.1 (A)
Cai et al. (2018)	Species presence	DNN	95.0 (A)
Nogueira et al. (2019)	Species presence	Cust. CNN	99.8 (A)

*Primary studies report performance as accuracy (A) and F-score (F)*.

**Regression problems**. CNN models are mainly used for two-dimensional arrays like image data. However, we can also apply CNN with regression data analysis. A regression layer can be included at the end of the network to predict continuous data, such as yield or days in the year. Three primary studies used deep learning techniques to solve a regression problem (cp. Table 7). Xin et al. (2020) retrieved phenological metrics from a time series of satellite data using a four-layer neural network. Their study was the only to directly predict the phenological events SOS and EOS from satellite images. Their results show that machine learning outperforms rule-based methods, although the authors noted that a random forest algorithm achieves better results than the neural network. Cao et al. (2021) developed a method to predict leaf phenology of deciduous broadleaf forests from individual PhenoCam images using deep learning approaches. They tested four convolutional network architectures (AlexNet-R, VGG-R, ResNet50-R, and ResNet101-R) for their ability to predict vegetation growing dates based on PhenoCam images at 56 different sites. In terms of model performance, ResNet achieved the best accuracy with an RMSE of 4.4 days. Yang et al. (2019) extracted 256 features from RGB images depicting the scene as well as 128 features from multispectral images depicting the same scene. The outputs of these two branches were concatenated into a feature vector and then fed into three consecutive fully connected layers to estimate yield at different phenological stages with a deep regressive approach. Deep learning with different training and testing strategies outperformed the traditional vegetation indices in all their experiments.

**Table 7:** Overview of deep learning methods for regression problems.

**Primary study**	**Regression task**	**Network architecture**	**Comapared methods**	**Performance as RMSE**
Xin et al. (2020)	SOS, EOS	Four-layer NN	**RF**, rule based methods	22.9, **21.5**, 27.30 days
Cao et al. (2021)	Leaf phenology	**ResNet50, ResNet101**	AlexNet, VGG	**4.4**, **4.4**, 7.6, 6.5 days
Yang et al. (2019)	Yield estimation	**Cust. two-branch CNN processing RGB and multispectral images**	Vegetation indices	**0.6** , 0.9 ton/ha

*Primary studies report performance as root mean square error (RMSE). The bold values indicate the best performing method and their respective performance*.

**Figure 8:**
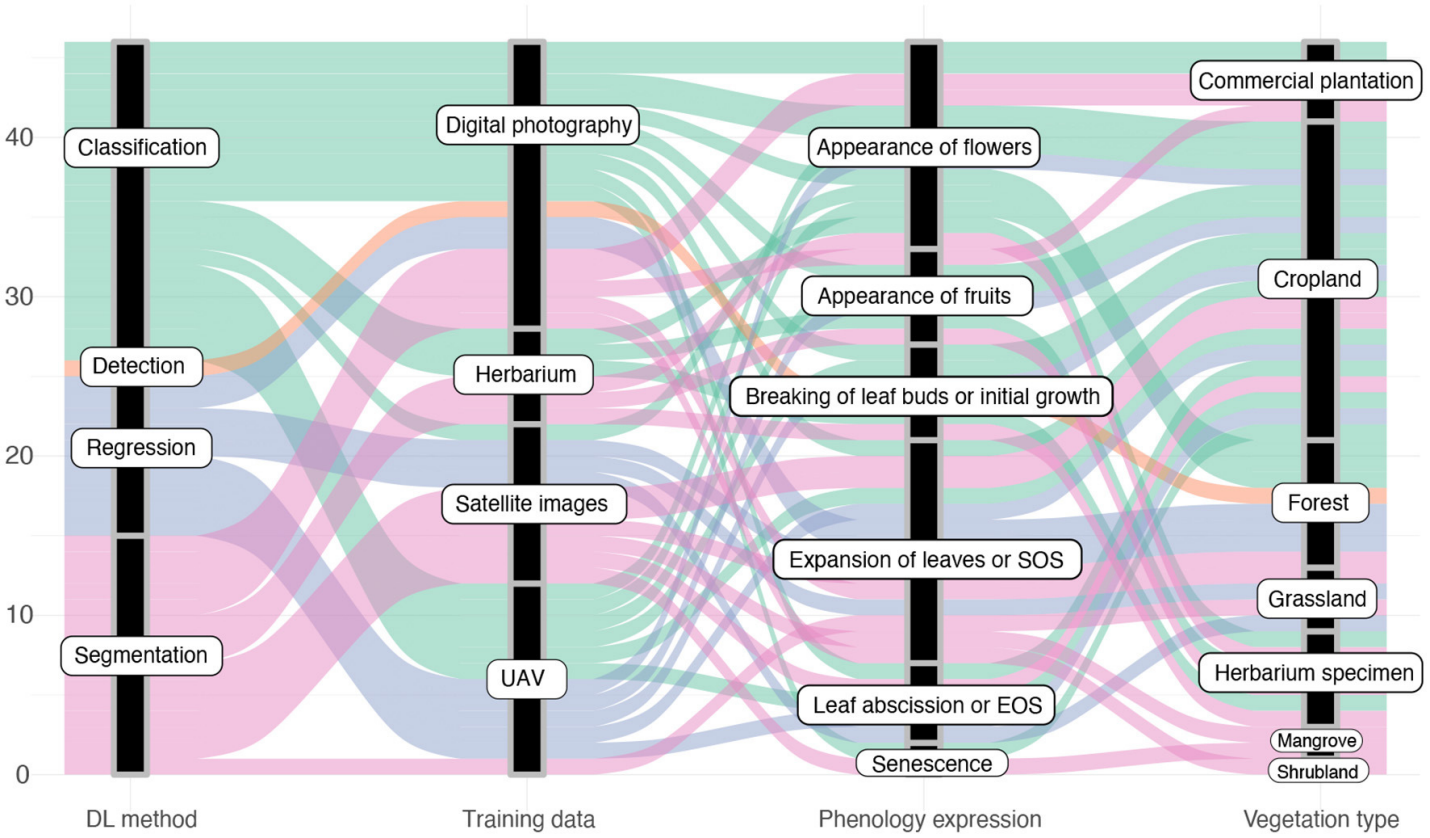
Diagram describing articles according to the defined categories.

In summary, the use of deep neural networks to analyze phenology imagery proofed very beneficial across a variety of studies. Researchers experimented with their capability in substituting tedious and error-prone manual tasks, as well as improving traditional analyses performed with shallow learning networks, conventional statistical methods, or *via* vegetational indices. In general, the reviewed primary studies showed patterns in how they applied DL methods, what type of training images they utilized, the type of studied phenology expression and the studied vegetation type. Figure 8 visualizes these patterns as a diagram. In particular, the following observations can be extracted from this diagram:

- The majority of DL approaches study segmentation and classification tasks.
- There has only been proposed one detection approach.
- Most of the used training images were acquired by near surface digital cameras.
- The most studied part of the phenology cycle was the spring aspect.
- Multiple phenological stages were especially studied on croplands, while in forest and plantation mostly one or two phenological stages were studied.

## 5. Research Trends and Future Direction

### 5.1. Automating Ground Based Observation

Increasing the number of ground-based observations is extremely important, especially in countries that lack traditional phenological observing networks. Most existing networks, aside from those in Europe, selected locations in North America, and a few other countries, have a relatively short historical record and often a limited number of stable, consistent monitoring locations. Building a global systematic, long-term monitoring network with unified standards of phenology measures and definitions is essential (Tang et al., 2016). The analysed primary studies in this review show that automated digital repeated photography combined with deep learning technologies for automated image-based phenology stage identification allows phenology monitoring similar to human-based observation. Such installations represent an extraordinary opportunity for phenology projects, like the US National Phenology Network or the European Phenology Network, and countries currently lacking a phenological monitoring network. With standardized recording systems and evaluation methods, ground-based phenological data can be recorded over larger areas in the future. Such data are essential for better understanding and predicting future environmental processes. In addition, ground-based camera systems and automated image analysis can provide high temporal resolution for calibrating satellite-based monitoring initiatives.

It is crucial to test the potentials and limits of different recording methods under different environmental conditions in this research area. In order to be able to evaluate the images created automatically, the recording situation is of great importance. So far, the focus has been on comparing different DL architectures and less on the image acquisition methods. Similar to experiments for automatic plant identification (Unger et al., 2016; Carranza-Rojas et al., 2017; Rzanny et al., 2017, 2019) experiments should also be carried out on different recording conditions for automated ground-based phenology stage monitoring. In addition, most studies so far have only focused on one phenological stage. It is essential to carry out phenological monitoring throughout the year. Therefore, in the future, models that allow monitoring different phenological stages throughout the year should be built and tested.

### 5.2. Analyzing Images From PhenoCam Networks With Deep Learning

The PhenoCam network provides free, publicly accessible digital imagery at the continental level. To date, most studies use indirect methods to identify phenology variation based on the time series of PhenoCam images. The indirect methods track changes in images by deriving handcrafted features such as green or red chromatic coordinates from PhenoCam images and then apply algorithms to derive the timing of phenological events, such as SOS and EOS. The use of these handcrafted features ignores high-level information in digital images. More importantly, these studies generally required at least two images or even the time series to obtain information related to leaf phenology. The study by Cao et al. (2021) was the first to directly monitor phenological events using deep learning technology and Phenocam Images. They tested deep learning models on predicting leaf growing dates after SOS in a year from a given PhenoCam image. Compared with traditional methods that predict leaf phenology with the mentioned handcrafted features from time-series data, they argued that the use of deep learning methods allows inferring daily leaf phenology from individual PhenoCam images and can potentially improve image processing accuracy and reduce laboratory costs. We expect many more studies to appear in the future evaluationg PhenoCam images beyond the vegetation color indices calculated so far.

### 5.3. Analysing Citizen Science Image Data With Deep Learning

An alternative set of resources that yet has to be harnessed for phenology studies comprises repositories of citizen science images. Citizen ccientists submit several thousand plant images daily collected with Apps like iNaturalist (Nugent, 2018), Pl@ntNet (Goëau et al., 2014) or Flora Incognita (Mäder et al., 2021). These images have a timestamp and location information and can thus provide important information about, e.g., flowering periods, similar to herbarium material. In a first study (Barve et al., 2020) introduced a method using Yucca flowering phenology as a case study for analyzing flowering phenology from iNaturalist images. In this study, however, the phenological annotation of the images was done manually which is very time-consuming and requires expert knowledge. A first, recently published study shows deep learning technologies can be successfully used to extract phenological information from citizen science images. A CNN classified a two-stage phenology (flowering and non-flowering) with 95.9% accuracy and a four-stage phenology (vegetative, budding, flowering, and fruiting) with 86.4% accuracy based on *Alliaria petiolata* (Reeb et al., 2022). As also the studies on the herbarium specimens showed, such annotations can also be done automatically with deep learning algorithms. Large image databases such as GBIF (www.gbif.org) and DigBio (www.idigbio.org) provide an excellent database for developing classifiers that not only automatically identify species (Carranza-Rojas et al., 2017; Wäldchen and Mäder, 2018a) but also phenological stages. If citizen scientists simply upload images of specific plants depicting different phenological stages, the obvious synergies between the large amounts of data generated by citizen science projects and the data-demanding analytical power of artificial intelligence could effectively be exploited (Correia et al., 2020).

### 5.4. Deep Learning for Phenology Modeling

Researchers have developed statistical and process-based models for forecasting the occurrence of vegetation phenological events. The idea of these modeling approaches is to use external climate data as input to predict the timing of key phenology metrics (Zhao et al., 2013; Hufkens et al., 2018). So far, phenology in the land surface models or dynamic global vegetation models (e.g., Biome-BGC (BioGeochemical Cycles) model, Lund-Potsdam-Jena model) generally adopt simple rule-based functions to account for the impacts of meteorological drivers, which can lead to large uncertainties in the modeled terrestrial ecosystem processes (Zhou et al., 2021). With the increase in automatically generated data (e.g., digital repeated photography in PhenoCam networks or satellite images), the amount of data available is constantly growing. It is time to explore new approaches on processing big data for phenology modeling. Only recently a study was published where a one-dimensional convolutional neural network regression (1D-CNNR) model was developed to model global vegetation phenology using meteorological variables and satellite images as inputs. This research by (Zhou et al., 2021) demonstrates that the 1D-CNNR model has the potential for large-scale modeling of vegetation phenology. Future research should integrate deep learning techniques even more into phenology modeling. Hybrid modeling approaches, that couple physical process models with the versatility of data-driven deep learning models, are a most promising future research direction (Reichstein et al., 2019).

## 6. Conclusions

Our review provides a comprehensive overview on the status and development of a recently emerging research field: the utilization of deep learning methods in plant phenology research. We describe and briefly summarize the extensive range of methods applied on different spatial and temporal scales concerning data collection and analysis. Altogether, we identified 24 studies meeting our criteria, most of them published in 2020/21. This review indicates that analysing phenology data with deep learning techniques is still in its infancy, compared to other fields such as, e.g., image-based automated species identification, where DL is already indispensable. Given the great potential for improving land surface- or dynamic vegetation models, it is time to develop standardized approaches for different scales and types of input data. So far, for the individual scale, the main focus of the studies was on recognizing phenological stages from images, while only very few monitored these stages over an entire year. However, for satellite and UAV-derived data, the focus was on identifying and localizing plant species based on species-specific phenological characteristics. A wide range of different DL methods was applied in the examined studies, with classification and segmentations being most often employed. Our finding highlights the great potential of DL to take plant phenology research to the next level, and we strongly encourage researchers to realize the enormous potential of DL methods in this research field.

## Data Availability Statement

The original contributions presented in the study are included in the article/Supplementary Material, further inquiries can be directed to the corresponding author/s.

## Author Contributions

NK, JW, MR, and PM: study conception and design. NK: performing literature search. NK and JW: analysis and interpretation of results, visualization, and writing manuscript. MR and PM: critical feedback and improvement. PM and JW: funding. All authors have approved the manuscript.

## Funding

This study was funded by the German Ministry of Education and Research (BMBF) grant: 01IS20062, the German Federal Ministry for the Environment, Nature Conservation, Building and Nuclear Safety (BMUB) grants: 3519685A08 and 3519685B08, and the Thuringian Ministry for Environment, Energy and Nature Conservation grant: 0901-44-8652.

## Conflict of Interest

The authors declare that the research was conducted in the absence of any commercial or financial relationships that could be construed as a potential conflict of interest.

## Publisher's Note

All claims expressed in this article are solely those of the authors and do not necessarily represent those of their affiliated organizations, or those of the publisher, the editors and the reviewers. Any product that may be evaluated in this article, or claim that may be made by its manufacturer, is not guaranteed or endorsed by the publisher.
